# Supplementary material for: Estrogen-sensitive medial preoptic area neurons coordinate torpor in mice
Source: Nat Commun. 2020 Dec 11;11:6378. doi: 10.1038/s41467-020-20050-1 (PMC7732979; doi:10.1038/s41467-020-20050-1)
Supplement: Supplementary file 1 — Supplementary Information [file 41467_2020_20050_MOESM1_ESM.pdf]

Supplementary Information

Estrogen-Sensitive Medial Preoptic Area Neurons Coordinate Torpor in Mice

Zhi Zhang[1,2], Fernando M. C. V. Reis[3], Yanlin He[4,5], Jae W. Park[1], Johnathon R. DiVittorio[1], Nilla Sivakumar[1], J. Edward van Veen[1,2], Sandra Maesta-Pereira[3], Michael Shum[6,7], India Nichols[1], Megan G. Massa[1,2], Shawn Anderson[1], Ketema Paul[1], Marc Liesa[6,7], Olujimi A. Ajijola[8], Yong Xu[4], Avishek Adhikari[2,3], Stephanie M. Correa[1,2]*

Affiliations
[1] Department of Integrative Biology and Physiology, University of California Los Angeles, CA, USA
[2] Brain Research Institute, University of California Los Angeles, CA, USA
[3] Department of Psychology, University of California Los Angeles, CA, USA
[4] USDA/ARS Children's Nutrition Research Center, Department of Pediatrics, Baylor College of Medicine, Houston, TX, USA
[5] Pennington Biomedical Research Center, Louisiana State University, Baton Rouge, LA, USA.
[6] Division of Endocrinology, Department of Medicine, David Geffen School of Medicine, Los Angeles, CA, USA
[7] Department of Molecular and Medical Pharmacology, David Geffen School of Medicine, Los Angeles, CA, USA
[8] UCLA Cardiac Arrhythmia Center, Department of Medicine, David Geffen School of Medicine, Los Angeles, CA, USA

*author for correspondence: stephaniecorrea@ucla.edu

Supplementary Information provided in this file:

Supplementary Figure 1. Activating ERα+ MPA neurons alters temperature and heat loss
Supplementary Figure 2. Chemogenetic activation of ERα+ MPA neurons induces a torpor-like state
Supplementary Figure 3. Neuronal activity of ERα+ MPA neurons during temperature challenge
Supplementary Figure 4. Sex differences after ablating ERα+ MPA cells in baseline activity and fasting-induced torpor
Supplementary Figure 5. Projections of ERα+ neurons from the MPA
Supplementary Table 1. qPCR primer sequences

Supplementary Files available separately:

Source_Data.xlsx

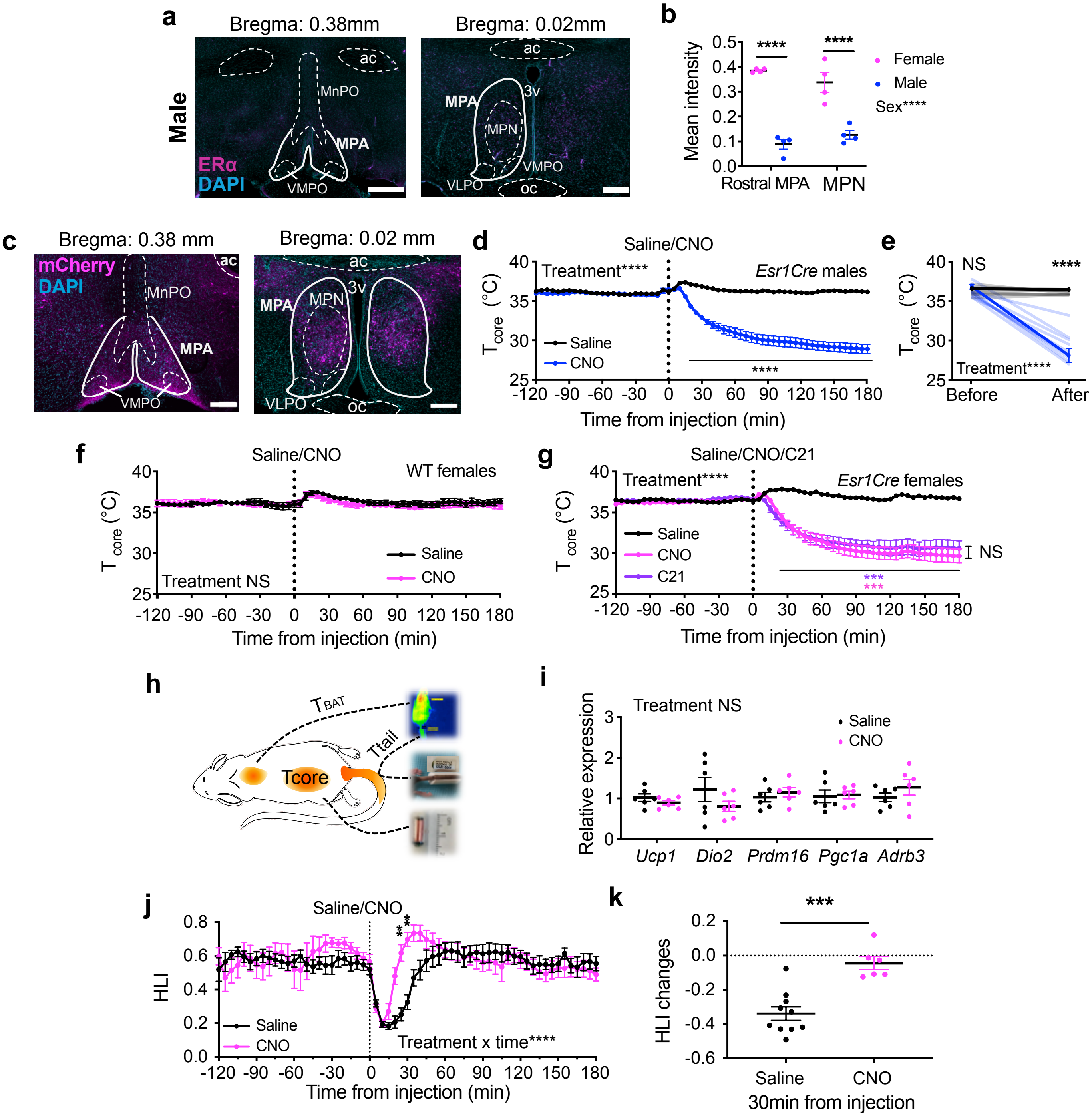

Supplementary Figure 1. Activating ERα+ MPA neurons alters temperature and heat loss. a, ERα immunoreactivity (magenta) in the MPA of adult male mice. Image representative of n = 4 mice. Scale bar: 200 µm. 3v, third ventricle; ac, anterior commissure; MnPO, median preoptic nucleus; MPA, medial preoptic area; MPN, medial preoptic nucleus; oc, optic chiasm; VLPO, ventrolateral preoptic nucleus; VMPO, ventromedial preoptic nucleus. **b,** Quantification of mean ERα intensity in rostral MPA and MPN; **c,** mCherry reporter expression in the MPA of *Esr1Cre* males following stereotaxic delivery of AAV-flex-hM3Dq-mCherry to the MPA. Images representative of n=9 mice. Scale bar: 200 µm. **d,** Core body temperature measured every 5 minutes before and after injection (dotted line at x=0) of saline (black) or CNO (blue) in n=9 *Esr1*Cre male mice. **e,** Per-animal averages of core temperature before (-120 min to 0 min) and after (120 min to 180 min) saline or CNO injection for animals shown in d. **f,** Core body temperature before and after injection of saline (black) or CNO (pink) in wide-type females (n=6). **g**, Core body temperature before and after injection of saline (black), CNO (pink) or C21

(purple) in *Esr1Cre* females (n=6). **h**, Schematic showing the strategy of measuring core (T_{core}), brown adipose tissue (T_{BAT}) and tail (T_{tail}) temperature. **i**, qPCR analysis of thermogenic gene expression in BAT 90 min after saline or CNO injection in *Esr1Cre* females expressing hM3Dq in the MPA (n=6 per group). **j**, heat loss index (HLI) calculated by attached tail thermo-logger before and after injection (dotted line at x = 0) of saline (black, n=10 mice) or CNO (pink, n=6 mice) in *Esr1Cre* females expressing hM3Dq in the MPA. **k**, Changes in HLI from baseline (-120 min to 0 min) to after (25 min to 35 min) saline or CNO injection. Statistical significance denoted by NS, not significant; **, p<0.05; ***, p<0.01; ****, p<0.0001 for Sidak's multiple comparison tests following a significant effect of treatment or the interaction of treatment with region **(b)** time **(d-g, i)** or gene **(h)** in a two-way ANOVA (**b, i**) or RM ANOVA **(d-g, j)**. ***, p<0.001 for (**k**) paired, two-tailed student's t-test. All error bars show SEM.

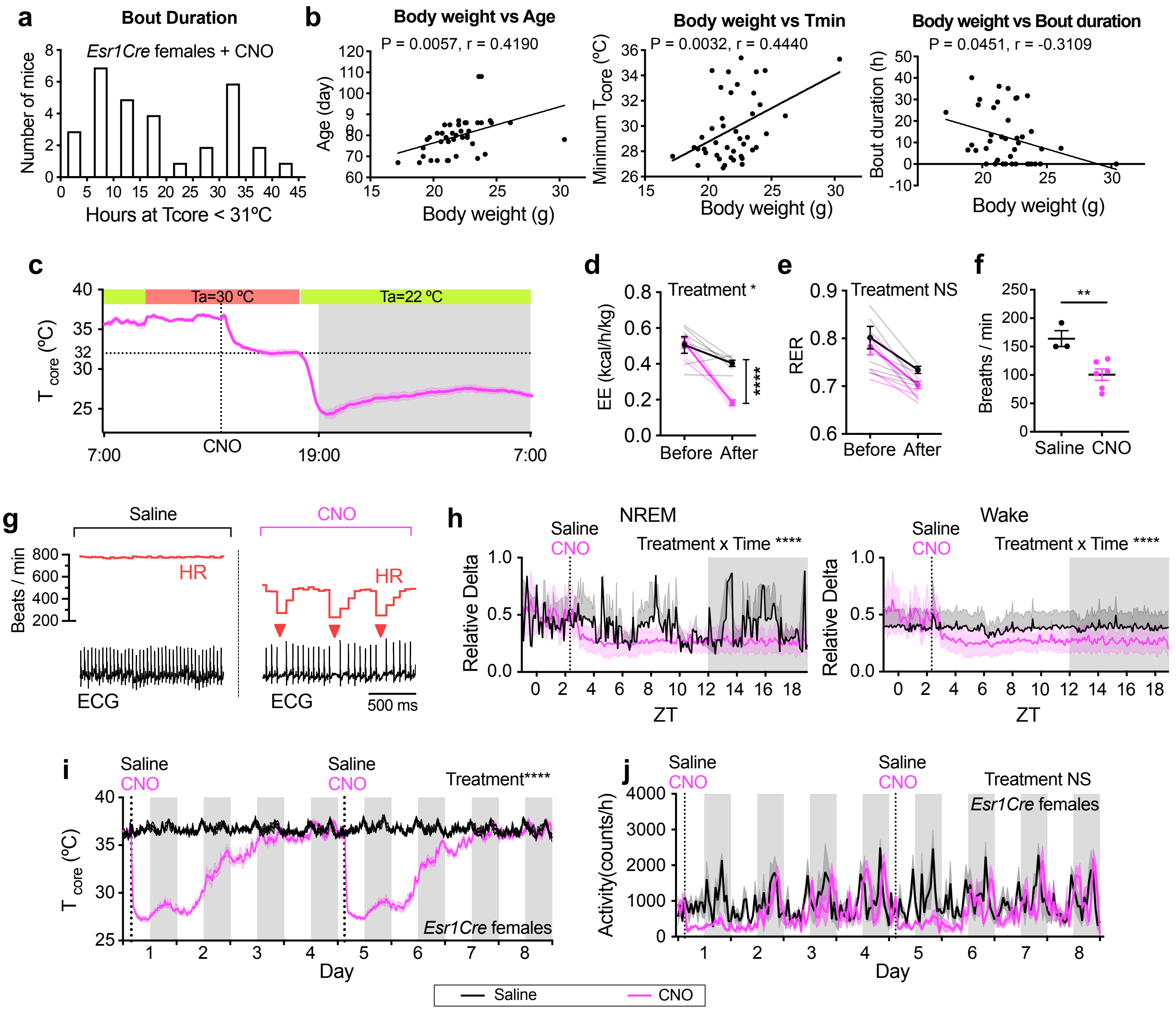

Supplementary Figure 2. Chemogenetic activation of ERα+ MPA neurons induces a torpor-like state. **a,** Frequency distribution showing duration of hypothermia bouts (Tcore < 31°C) after CNO injection (n=31 mice). **b,** Linear regressions for body weight vs. age, body weight vs. minimum core temperature (Tmin), and BW vs. bout duration after CNO injection (n=42 female *Esr1Cre* mice). **c,** Core temperature after CNO injection in mice (n=4 *Esr1Cre* females) during exposure to an ambient temperature (Ta) of 30°C (red bar) then 22°C (green bar). **d, e,** Energy expenditure (EE or heat generation kcal/h/kg) (**d**) and respiratory exchange ratio (RER) (**e**) before (-40 min to 0) and after (120 min to 180 min) saline or CNO injection calculated from indirect calorimetry data and normalized to lean body mass (n = 6 *Esr1Cre* females). **f,** Respiratory rate (breaths/min) quantified from >1 min videos of *Esr1Cre* mice 3 hours after saline (n = 3 females) or CNO (n = 5 females, 1 male) injection. **g,** Representative ECG in mice 4 h after saline or CNO injection. Red arrowheads indicate skipped heart beats. **h,** Relative delta power from 4-day consecutive EEG recordings with saline NREM vs. CNO and saline wake vs. CNO. White and grey bars represent light and dark periods. ZT, zeitgeber time. **i-j,** Eight-day consecutive recordings of core temperature (**i**) and movement (**j**) after two single injections of CNO (dotted line) in a cohort of *Esr1Cre* female mice showing long torpor bouts (n = 4 *Esr1Cre* females). Lines show group means, error bars or shaded area (**c, h-j**) show SEM, faded lines (**d, e**) show individual animals. ****, p<0.0001 for Sidak's multiple comparison tests comparing saline and CNO following a significant effect of treatment (**d** and **i**) in a two-way RM ANOVA or the interaction between treatment and time in a linear mixed effects model (**h**). **, p<0.01 for unpaired, 2-tailed student's t-test (**f**).

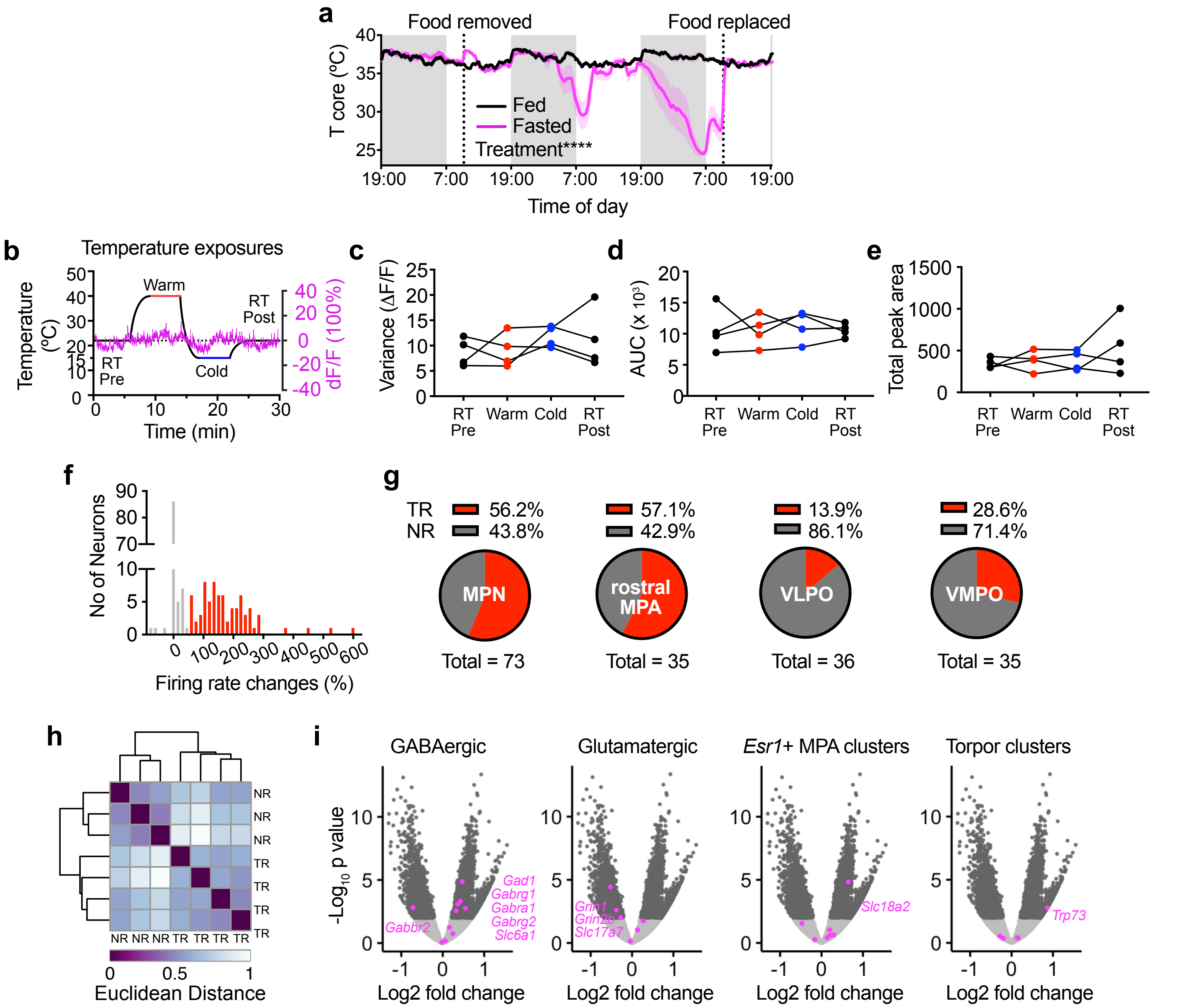

Supplementary Figure 3. Neuronal activity of ERα+ MPA neurons during temperature challenge. **a,** Fasting induced hypothermia as model for torpor (n=6, females). Shaded areas show SEM. **b,** Representative trace recorded during gradual warm (40 °C) and cold (15 °C) temperature exposure. **c-e,** Individual mouse responses during each temperature exposure period (5 min), including variance (**c**), area under the curve, AUC (**d**) and total peak area (**e**). **f,** Distribution of neurons by firing rate changes in response to temperature change. Neurons showing <=41.4% change in firing rate were classified as non-responsive (NR, grey) and neurons showing >41.4% change in firing rate were classified as temperature responsive (TR, red). n = 179 cells from 4 female mice. **g,** Pie charts showing percentages of TR (red) and NR (grey) neurons in the medial preoptic nucleus, (MPN), rostral MPA, ventrolateral preoptic nucleus (VLPO), and ventromedial preoptic nucleus (VMPO). **h,** Heat map comparing transcriptomes of NR (n = 3 samples, two neurons per sample) or TR (n = 4 samples, 2 neurons per sample) neurons when exposed to 25 °C and 30 °C. **i**, Volcano plots comparing gene expression in TR versus NR ERα neurons in the MPN. All genes shown as grey dots, those with significant differential expression (adjusted p<0.05 using the Benjamini-Hochberg procedure) denoted by dark grey dots, and transcripts of interest denoted by pink dots. Transcripts of interest include markers of GABAergic neurons, glutamatergic neurons, distinct *Esr1*-expressing clusters in the MPA (from Ref. 14), and markers of neurons activated by torpor (from Ref. 39). Gene symbols are provided for genes that are significantly enriched (right) or depleted (left) in TR ERα neurons. Genes that were detected in TR and NR but not differentially expressed in TR neurons include *Gabra2, Gabbr1, Gad2, Slc32a1* for GABAergic markers, *Slc17a6, Gls, Glul* for glutamatergic markers, *Trhr, Nts, Calcr, Tac2, Etv1, Mc4r* for *Esr1* cluster markers, and *Sntg2*, *Nts*, *Pde1c*, *Map3k15* for torpor neuron markers.

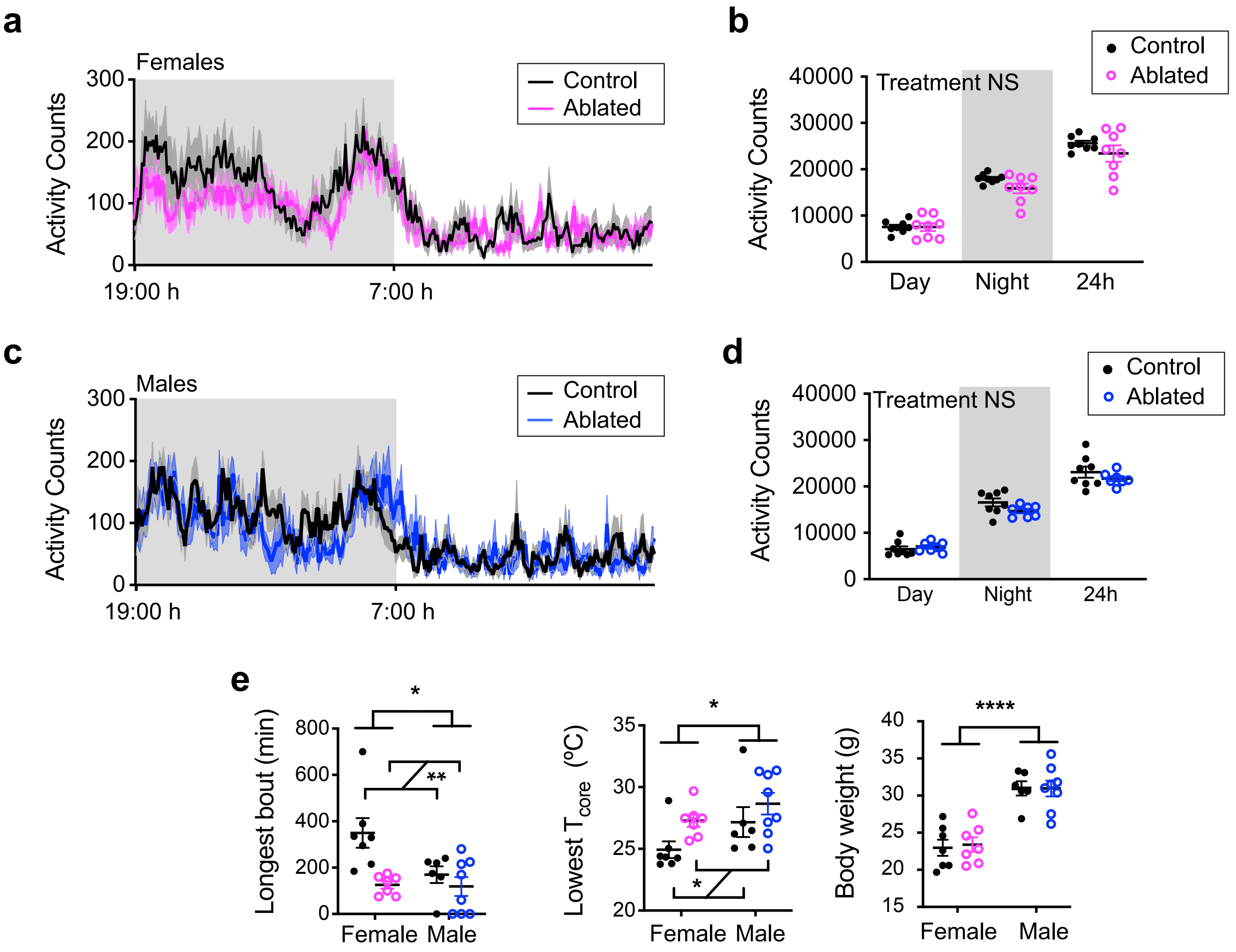

Supplementary Figure 4. Sex differences after ablating ERα+ MPA cells in baseline activity and fasting-induced torpor. a and **c,** Physical activity over 24 h, measured every 5 min for 3 days. Group averages shown for control (black, n=8) and ablated (pink, n=8 female; blue, n=7 male) mice. Shading along the curve denotes the SEM. **b** and **d**, Sum of activity from mice shown in panel **a** and **c** highlighting per animal in light (7:00 to 19:00), dark (19:00 to 7:00), and total 24 h periods. **e**, Duration of the longest bout of Tcore ≤ 31 °C, lowest Tcore and body weight during the fasting period for control females (n=7 mice), ablated females (n=7 mice), control males (n=6 mice), and ablated males (n=8 mice). Error bars show SEM. Statistical significance denoted by NS, not significant effect of treatment in a two-way RM ANOVA (**b** and **d**), *, p<0.05 and **, p<0.01, ****, p<0.0001 for two-way ANOVA (**e**).

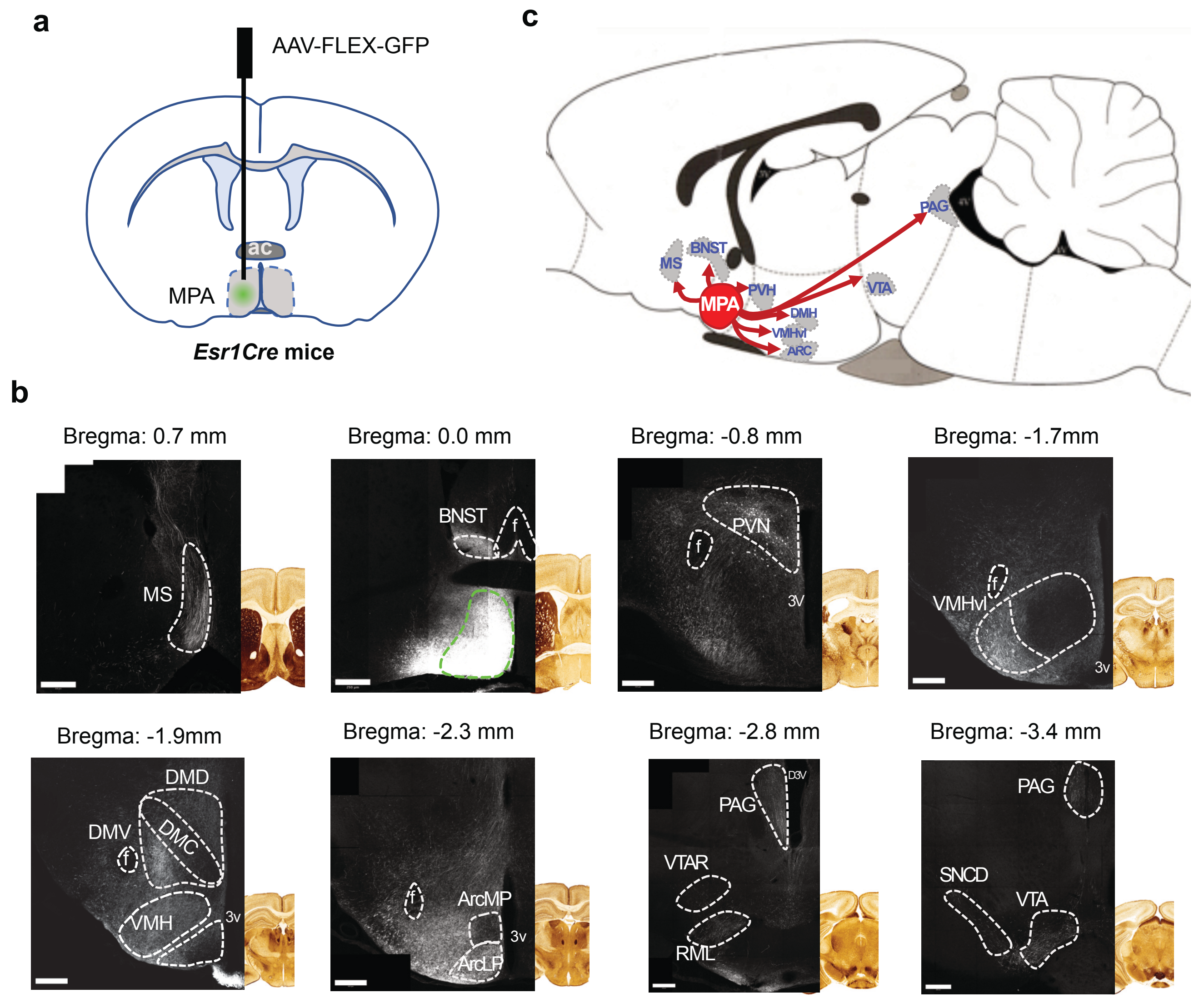

Supplementary Figure 5. Projections of ERα+ neurons from the MPA. a, Schematic strategy for visualizing descending target structures from the MPA using Cre-dependent virus expressing GFP. **b**, GFP expression in ERα+ cell bodies at the injection site (green dashed outline in Bregma: 0.0 mm) and in fibers from the major projection targets. Projection profile is shown 2 weeks after GFP injection. Images representative of n=3 *Esr1Cre* female mice. 3v, third ventricle; ac, anterior commissure; ArcMP or ArcLP, arcuate nucleus, medial posterior part (ArcMP) or lateroposterior part (ArcLP); BNST, bed nucleus of stria terminalis; f, fornix; D3V, dorsal third ventricle; DMC (DMD/DMV), dorsal medial hypothalamus, compact part (or dorsal/ventral parts); MPA, medial preoptic area; MS, medial septum nucleus; PAG, periaqueductal gray; PVH, paraventricular hypothalamus; RML, retromammillary nucleus, lateral part; SNCD, substantia nigra, compact part, dorsal tier; VMH (vl), ventromedial hypothalamus (ventral lateral part); VTA (R), ventral tegmental area (rostral part). Scale bars, 250μm. **c**, Schematic summary of the major projection brain areas from ERα+ MPA neurons.

Supplementary Table 1. qPCR primer sequences

Gene name	Symbol	Forward	Reverse
Uncoupling protein 1	*Ucp1*	CACGGGGACCTACAATGCTT	TAGGGGTCGTCCCTTTCCAA
Deiodinase, iodothyronine, type II	*Dio2*	CCTCAGAAGGGCTGCGCTGTG	TCAGCGGTCTTCTCCGAGGC
PR domain containing 16	*Prdm16*	GAAGTCACAGGAGGACACGG	TCATTGCATATGCCTCCGGG
Peroxisome proliferator-activated receptor gamma, coactivator 1 alpha	*Pgc1a*	CAGTACAGCCCCGATGACTC	GAAAGCTCGTCCACGTCAGAC
Adrenergic receptor, beta 3	*Adrb3*	GGAAGCTTGCTTGATCCCCA	GCCGTTGCTTGTCTTTCTGG
